# Supplementary material for: Outbreaks of publications about emerging infectious diseases: the case of SARS-CoV-2 and Zika virus
Source: BMC Med Res Methodol. 2021 Mar 11;21:50. doi: 10.1186/s12874-021-01244-7 (PMC7948668; doi:10.1186/s12874-021-01244-7)
Supplement: Supplementary file 1 — Additional file 1: Supplementary Text 1. Search strategy.Supplementary Table S2. Number and proportion of publications on Zika virus by category and publication type.Supplementary Table S3. Number and proportion of publications on SARS-CoV-2 by category and publication type.Supplementary Figure S1. Proportion of different study designs of published research on SARS-CoV-2 (SARS-CoV-2) and Zika virus (ZIKV) over time, without preprint publications.Supplementary Figure S2. Number of different study designs of published research on SARS-CoV-2 (SARS-CoV-2) and Zika virus (ZIKV) over time.Supplementary Figure S3. Time to the first 20 publications in a study design, for SARS-CoV-2 infections (SARS-CoV-2) and Zika virus infections (Zika virus), excluding preprint publications.Supplementary Figure S4. The proportion of preprint publications compared to and peer-reviewed publications for SARS-CoV-2 by study design and week. [file 12874_2021_1244_MOESM1_ESM.docx]

# Additional file 1

“Outbreaks of publications about emerging infectious diseases: the case of

SARS-CoV-2 and Zika virus”

Aziz Mert Ipekci, Diana Buitrago-Garcia, Kaspar Walter Meili, Fabienne Krauer, Nirmala Prajapati, Shabnam

Thapa, Lea Wildisen, Lucia Araujo Chaveron, Lukas Baumann, Sanam Shah, Tessa Whiteley, Gonzalo Solís-

García, Foteini Tsotra, Ivan Zhelyazkov, Hira Imeri, Nicola Low, Michel Jacques Counotte

**Table of Contents**

[Additional file 1	1](#_Toc64459378)

[**Supplementary Text 1. Search strategy**	2](#_Toc64459379)

[**Supplementary Table S2. Number and proportion of publications on Zika virus by category and publication type.**	3](#_Toc64459380)

[**Supplementary Table S3. Number and proportion of publications on SARS-CoV-2 by category and publication type.**	4](#_Toc64459381)

[**Supplementary Figure S1. Proportion of different study designs of published research on SARS-CoV-2 (SARS-CoV-2) and Zika virus (ZIKV) over time, without preprint publications.**	5](#_Toc64459382)

[**Supplementary Figure S2. Number of different study designs of published research on SARS-CoV-2 (SARS-CoV-2) and Zika virus (ZIKV) over time.**	6](#_Toc64459383)

[**Supplementary Figure S3. Time to the first 20 publications in a study design, for SARS-CoV-2 infections (SARS-CoV-2) and Zika virus infections (Zika virus), excluding preprint publications.**	7](#_Toc64459384)

[**Supplementary Figure S4. The proportion of preprint publications compared to and peer-reviewed publications for SARS-CoV-2 by study design and week.**	8](#_Toc64459385)

## **Supplementary Text 1. Search strategy**

**COVID:**

EMBASE:

(SARS coronavirus/ OR middle east respiratory syndrome/ OR severe acute respiratory syndrome/ OR (coronavirus* OR corona virus* OR HCoV* or ncov* OR covid or covid19 OR sars-cov* or sarscov* or Sars-coronavirus* OR Severe Acute Respiratory Syndrome Coronavirus*).mp.) and 20191201:20301231.(dc).

MEDLINE:

("coronavirus"[MH] OR "coronavirus infections"[MH] OR "coronavirus"[TW] OR "corona virus"[TW] OR "HCoV"[TW] OR "nCov"[TW] OR "covid"[TW] OR "covid19"[TW] OR "Severe Acute Respiratory Syndrome Coronavirus 2"[TW] OR "SARS-CoV2"[TW] OR "SARS-CoV 2"[TW] OR "SARS Coronavirus 2"[TW] OR "MERS-CoV"[TW]) AND (2019/1/1:3000[PDAT])

From 01.04.2020, we retrieve the currate BioRxiv/MedRxiv dataset [<https://connect.medrxiv.org/relate/content/181> ]

<https://ispmbern.github.io/covid-19/living-review/collectingdata.html>

**Zika:** All sources: Zika OR ZIKV OR “Zika virus”

Additional information is provided here: <https://journals.plos.org/plosmedicine/article?id=10.1371/journal.pmed.1002203#pmed.1002203.s002>

## **Supplementary Table S2. Number and proportion of publications on Zika virus by category and publication type.**

|  | **Preprint (N=98)** | **Peer-reviewed (N=2188)** | **Overall (N=2286)** |
| --- | --- | --- | --- |
| **Study design group** |  |  |  |
| Epi | 31 (31.6%) | 521 (23.8%) | 552 (24.1%) |
| Basic | 37 (37.8%) | 215 (9.8%) | 252 (11.0%) |
| Other | 30 (30.6%) | 1452 (66.4%) | 1482 (64.8%) |
| **Source** |  |  |  |
| MedRxiv | 0 (0%) | 0 (0%) | 0 (0%) |
| BioRxiv | 98 (100%) | 0 (0%) | 98 (4.3%) |
| EMBASE | 0 (0%) | 356 (16.3%) | 356 (15.6%) |
| Pubmed | 0 (0%) | 1832 (83.7%) | 1832 (80.1%) |
| **Study design** |  |  |  |
| Case report | 1 (1.0%) | 128 (5.9%) | 129 (5.6%) |
| Case series | 2 (2.0%) | 92 (4.2%) | 94 (4.1%) |
| Case-control study | 0 (0%) | 5 (0.2%) | 5 (0.2%) |
| Cohort study | 0 (0%) | 13 (0.6%) | 13 (0.6%) |
| Cross-sectional study | 0 (0%) | 27 (1.2%) | 27 (1.2%) |
| Diagnostic study | 0 (0%) | 30 (1.4%) | 30 (1.3%) |
| Ecological study | 3 (3.1%) | 9 (0.4%) | 12 (0.5%) |
| Guidelines | 0 (0%) | 22 (1.0%) | 22 (1.0%) |
| Modelling study | 22 (22.4%) | 52 (2.4%) | 74 (3.2%) |
| Other | 6 (6.1%) | 37 (1.7%) | 43 (1.9%) |
| Outbreak or surveillance report | 0 (0%) | 33 (1.5%) | 33 (1.4%) |
| Qualitative study | 0 (0%) | 23 (1.1%) | 23 (1.0%) |
| Review | 0 (0%) | 82 (3.7%) | 82 (3.6%) |
| Trial | 0 (0%) | 1 (0.0%) | 1 (0.0%) |
| Animal experiment | 8 (8.2%) | 45 (2.1%) | 53 (2.3%) |
| In vitro experiment | 8 (8.2%) | 79 (3.6%) | 87 (3.8%) |
| Biochemical/protein structure studies | 10 (10.2%) | 62 (2.8%) | 72 (3.1%) |
| Sequencing and phylogenetics | 21 (21.4%) | 57 (2.6%) | 78 (3.4%) |
| Within-host modelling | 0 (0%) | 0 (0%) | 0 (0%) |
| Basic research review | 0 (0%) | 34 (1.6%) | 34 (1.5%) |
| Non-original | 8 (8.2%) | 1248 (57.0%) | 1256 (54.9%) |
| Mosquito | 9 (9.2%) | 109 (5.0%) | 118 (5.2%) |

## **Supplementary Table S3. Number and proportion of publications on SARS-CoV-2 by category and publication type.**

|  | **Preprint (N=1244)** | **Peer-reviewed (N=4050)** | **Overall (N=5294)** |
| --- | --- | --- | --- |
| **Study design group** |  |  |  |
| Epi | 909 (73.1%) | 1874 (46.3%) | 2783 (52.6%) |
| Basic | 194 (15.6%) | 218 (5.4%) | 412 (7.8%) |
| Other | 141 (11.3%) | 1958 (48.3%) | 2099 (39.6%) |
| **Source** |  |  |  |
| MedRxiv | 950 (76.4%) | 0 (0%) | 950 (17.9%) |
| BioRxiv | 294 (23.6%) | 0 (0%) | 294 (5.6%) |
| EMBASE | 0 (0%) | 676 (16.7%) | 676 (12.8%) |
| Pubmed | 0 (0%) | 3374 (83.3%) | 3374 (63.7%) |
| **Study design** |  |  |  |
| Case report | 5 (0.4%) | 231 (5.7%) | 236 (4.5%) |
| Case series | 67 (5.4%) | 264 (6.5%) | 331 (6.3%) |
| Case-control study | 13 (1.0%) | 24 (0.6%) | 37 (0.7%) |
| Cohort study | 70 (5.6%) | 105 (2.6%) | 175 (3.3%) |
| Cross-sectional study | 94 (7.6%) | 125 (3.1%) | 219 (4.1%) |
| Diagnostic study | 73 (5.9%) | 61 (1.5%) | 134 (2.5%) |
| Ecological study | 36 (2.9%) | 24 (0.6%) | 60 (1.1%) |
| Guidelines | 3 (0.2%) | 264 (6.5%) | 267 (5.0%) |
| Modelling study | 413 (33.2%) | 122 (3.0%) | 535 (10.1%) |
| Other | 45 (3.6%) | 166 (4.1%) | 211 (4.0%) |
| Outbreak or surveillance report | 23 (1.8%) | 78 (1.9%) | 101 (1.9%) |
| Qualitative study | 4 (0.3%) | 25 (0.6%) | 29 (0.5%) |
| Review | 66 (5.3%) | 475 (11.7%) | 541 (10.2%) |
| Trial | 17 (1.4%) | 10 (0.2%) | 27 (0.5%) |
| Animal experiment | 17 (1.4%) | 14 (0.3%) | 31 (0.6%) |
| In vitro experiment | 40 (3.2%) | 21 (0.5%) | 61 (1.2%) |
| Biochemical/protein structure studies | 116 (9.3%) | 66 (1.6%) | 182 (3.4%) |
| Sequencing and phylogenetics | 118 (9.5%) | 65 (1.6%) | 183 (3.5%) |
| Within-host modelling | 14 (1.1%) | 2 (0.0%) | 16 (0.3%) |
| Basic research review | 5 (0.4%) | 116 (2.9%) | 121 (2.3%) |
| Non-original | 5 (0.4%) | 1792 (44.2%) | 1797 (33.9%) |

## **Supplementary Figure S1. Proportion of different study designs of published research on SARS-CoV-2 (SARS-CoV-2) and Zika virus (ZIKV) over time, without preprint publications.**

Epidemiological (Epi), basic research and non-original research (A); Epidemiological and basic research by study design (B); epidemiological research by study design (C). PHEIC: Public Health Emergency of International Concern.

## **Supplementary Figure S2. Number of different study designs of published research on SARS-CoV-2 (SARS-CoV-2) and Zika virus (ZIKV) over time.**

Epidemiological (Epi), basic research and non-original research (A); Epidemiological and basic research by study design (B); epidemiological research by study design (C). PHEIC: Public Health Emergency of International Concern.

## **Supplementary Figure S3. Time to the first 20 publications in a study design, for SARS-CoV-2 infections (SARS-CoV-2) and Zika virus infections (Zika virus), excluding preprint publications.**

## **Supplementary Figure S4. The proportion of preprint publications compared to and peer-reviewed publications for SARS-CoV-2 by study design and week.**
